# The p75^NTR^-mediated effect of nerve growth factor in L6C5 myogenic cells

**DOI:** 10.1186/s13104-017-2994-x

**Published:** 2017-12-04

**Authors:** Alessandra de Perini, Ivan Dimauro, Guglielmo Duranti, Cristina Fantini, Neri Mercatelli, Roberta Ceci, Luigi Di Luigi, Stefania Sabatini, Daniela Caporossi

**Affiliations:** 1 Department of Movement, Human and Health Sciences, University of Rome Foro Italico, Piazza Lauro de Bosis, 15, 00135 Rome, Italy; 2 Laboratory of Cellular and Molecular Neurobiology, CERC, Fondazione Santa Lucia, Via del Fosso di Fiorano, 64, 00143 Rome, Italy

**Keywords:** NGF, p75^NTR^, Myogenic differentiation, Energy metabolism, NFκB, sHSPs

## Abstract

**Objective:**

During muscle development or regeneration, myocytes produce nerve growth factor (NGF) as well as its tyrosine-kinase and p75-neurotrophin (p75^NTR^) receptors. It has been published that the p75^NTR^ receptor could represent a key regulator of NGF-mediated myoprotective effect on satellite cells, but the precise function of NGF/p75 signaling pathway on myogenic cell proliferation, survival and differentiation remains fragmented and controversial. Here, we verified the role of NGF in the growth, survival and differentiation of p75^NTR^-expressing L6C5 myogenic cells, specifically inquiring for the putative involvement of the nuclear factor κB (NFκB) and the small heat shock proteins (sHSPs) αB-crystallin and Hsp27 in these processes.

**Results:**

Although NGF was not effective in modulating myogenic cell growth or survival in both standard or stress conditions, we demonstrated for the first time that, under serum deprivation, NGF sustained the activity of some key enzymes involved in energy metabolism. Moreover, we confirmed that NGF promotes myogenic fusion and expression of the structural protein myosin heavy chain while modulating NFκB activation and the content of sHSPs correlated with the differentiation process. We conclude that p75^NTR^ is sufficient to mediate the modulation of L6C5 myogenic differentiation by NGF in term of structural, metabolic and functional changes.

**Electronic supplementary material:**

The online version of this article (10.1186/s13104-017-2994-x) contains supplementary material, which is available to authorized users.

## Introduction

Skeletal muscle regeneration depends upon optimal activation, proliferation and differentiation of myogenic precursors, the satellite cells, whose behavior is controlled by components, such as cytokines and growth factors, contained in the satellite cell microenvironment [1]. During myogenic differentiation, muscle cells produce NGF and other neurotrophins as well as their receptors, tyrosine-kinase receptors (TrkA and TrkB) and p75-neurotrophin receptor (p75^NTR^), able to act in autocrine manner on cell morphology, proliferation and differentiation [2, 3]. Beside some contractictory results [4], several evidences demonstrate that cellular signaling pathways activated by the neurotrophin/p75^NTR^ axis stabilize the cytoskeletal architecture and increase the fusogenic properties of myotubes, thus promoting “in vitro” myogenic differentiation, myotubes survival and muscle repair “in vivo” [3, 5]. Thus, despite the well known pro-apoptotic role of p75^NTR^ in neuron cells, this receptor might represent a key mediator of survival in myoblasts and myotubes and its activity during myogenesis seems important for developing skeletal muscle [6, 7].

We previously demonstrated that the activation of NF-κB and the parallel modulation of αB-crystallin (αB-Cry) and Hsp27 play a major role in the antiapoptotic effect exerted by vascular endothelial growth factor (VEGF) in C2C12 myoblasts exposed to oxidative or hypoxic-like stress “in vitro” [8], as well as in the positive effect exerted by platelet-rich plasma on rat skeletal muscle healing “in vivo” [9]. NF-κB is a redox-sensitive transcription factor known to regulate several cellular processes (i.e. inflammation, cellular survival, proliferation and differentiation) that recently emerged as a key player in the regulation of skeletal muscle homeostasis [10]. αB-Cry and Hsp27 are small heat shock proteins (sHSPs) abundantly expressed in muscle tissue where they stabilize cytoskeletal structures, especially under pro-oxidant insult [11–13], modulate myogenic differentiation [14, 15] and interact with several growth factors through the c-Jun N-terminal kinase (JNK)- and/or NFκB- dependent regulatory mechanisms [9, 11, 12].

Considering these observations altogether, the main aim of the present study was to analyze in L6C5 myogenic cells expressing exclusively the p75^NTR^ receptor, whether the effect of NGF on myoblast growth, survival, fusion rate and expression of early and late markers of myogenesis correlates with NFκB activation and/or modulation of αB-Cry and Hsp27 expression.

## Main text

### Materials and methods

#### Cell culture, growth and viability

All experiments were carried out on L6C5 rat myogenic cells (ICLC, AL00001). As already described [16], L6C5 myoblast cultures were maintained and subcultured in growing medium (GM) (DMEM with 4.5 g/l glucose and Corning^®^ glutagro™, w/o sodium pyruvate, Corning 10-102-CVR; 100 U/ml penicillin, 100 μg/ml streptomycin, Euroclone ECB3001D; 10% FBS, Gibco 10270). Differentiation medium (DM) containing 2% FBS was utilized in 85%—confluent cells to induce myotubes. The process was monitored through microscopy and expression of myogenic markers [17, 18]. NGF (Promega, 10–100 ng/ml) was added to the GM or DM medium at the indicated time and replenished with media changes every 3 days. For cell growth and viability, 5 × 10^5^ cells/well were seeded in a 96-well culture plate for 6, 12, 24, and 48 h with or without 10% FBS, in presence or absence of NGF added from the seeding. Cell growth was analysed by MTS assay (Promega) following the manufacturer’s recommendations. The absorbance was measured at 490 nm (Bio-Rad680). Viability was evaluated by trypan blue exclusion assay performed at the same culture conditions.

#### Enzymatic activities

L6C5 myoblasts grown in presence or in absence of NGF (10, 100 ng/ml) for 48 h under standard (GM) or serum starvation conditions were lysed (0.05 M Tris–Acetate, 250 mM sucrose, pH 7.5, 1 mM PMSF) with a protease inhibitor cocktail (P8340, Sigma Aldrich, St. Louis, MO). After gentle sonication (twice 10 s in ice with Vibra-Cell CV 18 SONICS VX 11) and centrifugation (at 14,000 rpm for 10 min at 4 °C), the supernatant was tested for protein content (Bradford method, Sigma Aldrich, St Louis MO) and then analysed spectrophotometrically (20–50 µl sample, Perkin Elmer Lambda 25, Fremont, CA, USA) for glyceraldehyde-phosphate-dehydrogenase (GAPDH), lactate dehydrogenase (LDH), citrate synthase (CS), 3-OH acylCoA dehydrogenase (HAD) and alanine transglutaminase (ALT) enzymatic activities as previously described [19, 20].

#### Immunoblot analysis

Protein extraction and immunoblotting was performed by standard methodology as already described [9, 21]. Briefly, total cellular proteins were extracted by lysis buffer (20 mM Tris pH 7.5, 150 mM NaCl, 2 mM EDTA, 1 mM sodium orthovanadate, 100 mM PMSF, 10 mg/ml leupeptin, 10 mg/ml aprotinin, 5 mg/ml pepstatin, 50 mM NaF, 1 nM okadaic acid, 1% Triton X-100 and 10% glycerol), and quantified using the Bradford assay (Sigma). From each sample, 15–20 μg of proteins have been utilized for immunoblotting with the following antibodies: myogenin (sc-576), Hsp27 (sc-1048), p75^NTR^ (sc-56448) and TrkA (sc-20539) (1:1000, Santa-Cruz Biotechnology), β-actin (A1978) (1:3000, Sigma), anti-embryonic MyHC (F1652) (1:1000, Biovalley) and MyHC IIB (BFF3) (1:1000, Development Studies Hybridoma Bank), αB-crystallin (SPA-222) (1:1000, Enzo Life Sciences), SAPK/JNK (#9252), Phospho-SAPK/JNK (Thr183/Tyr185) (#4668), and Bcl-2 (#2870) (1:1000, Cell Signalling), Caspase 3 (#44976) (1:1000, Abcam). All immunoblots were visualized with the appropriated horseradish peroxidase-conjugated secondary antibody (1:15,000, Millipore) followed by detection with enhanced chemiluminescence (Amersham-Biosciences). Bands were quantified by ImageJ software. The expression of β-actin was used as a normalizing control.

#### Fusion rate analysis

Cells, grown 2 and 9 days in DM with or without NGF (20 ng/ml), were fixed with ice-cold methanol (7′ at − 20 °C), permeabilized (0.1% triton X-100 for 20′) and then blocked at RT for 60′ (TBS, 10% FBS, 0.1% triton). Myotubes were identified by double staining with Hoechst 33258 (Sigma) and MyHC antibody (sc-12117, 1:50, Santa-Cruz Biotechnology) as MyHC positive cells with at least two nuclei. Since the number of total nuclei was not modulated by NGF (data not shown), the fusion rate was evaluated by the number of total myotubes in each well and the number of nuclei/myotube [3].

#### NFκB activity

NFκB activity was measured in nuclear protein extracts (15 μg) by the TransAM NF-κB p65 protein assay (Active Motif), according to the manufacturer’s protocol [8]. The assay was performed in presence or in absence of NGF (20 ng/ml) on proliferating myoblasts (24 h from seeding) or cells grown in DM for 2, 5 and 9 days. Experimental samples and controls were run in duplicate.

#### Statistical analysis

Statistical comparisons between groups were performed by Student’s t test. All values are given as the mean ± standard deviation of the mean (SD). *p* < 0.05 was considered significant.

### Results and discussion

Impairment of the satellite cell pool has been observed in age-related muscle dysfunction and muscle degenerative pathologies, while the poor rate of survival and proliferation of myoblasts derived from satellite cell transplantation represents an important limit for the cell replacement therapy in muscle diseases [22]. Various components of the microenvironment play a crucial role in the proliferative and differentiation potential of satellite cells, thus ensuring an adequate regenerative response to muscle insult [1]. This study was indeed designed to confirm and extend the positive role of NGF/p75^NTR^ axis in “in vitro” differentiation of L6C5 myogenic cells.

As already described in other murine cell lines and in human muscle cells [3, 5–7], L6C5 cell line expressed only p75^NTR^ mRNA that, differently from primary myogenic cells [3], was not differentially expressed in myoblasts or during myogenesis, at both mRNA and protein levels (Additional file 1: Fig. S1a–c).

**Fig. 1:**
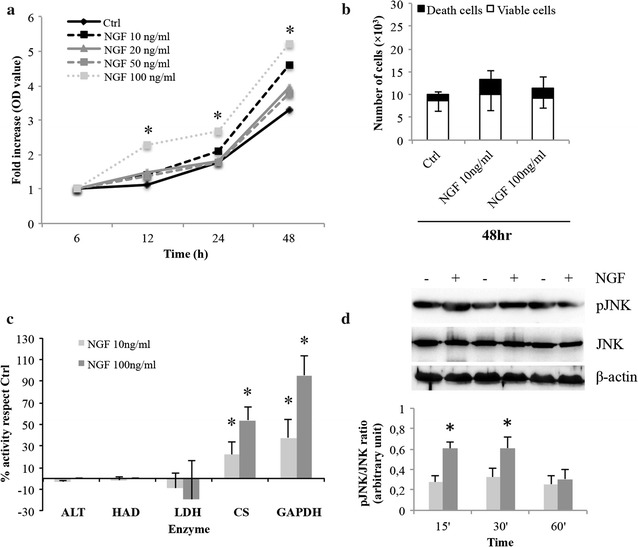
Cell growth, viability, enzymatic activities and JNK phosphorylation in L6C5 myoblasts under serum starvation condition. **a** MTS viability assay. Cell growth values are expressed as fold increase in absorbance (OD) values after normalization according to values obtained at 6 h from the seeding, when cells usually become adherent to the plate and start growing (NGF 100 ng/ml: 2.2- to 5.21-fold increase vs Ctrl: 1.1- to 3.27-fold increase, *p* < 0.05). **b** Trypan blue exclusion assay. Analysis of viable, trypan blue negative (white) and nonviable, trypan blue positive (black) cells was performed after 48 h with or without NGF (10 and 100 ng/ml) and it is expressed as total number of counted cells (Viable cells: Ctrl, 8.8 ± 2.5 × 10^3^; NGF 10 ng/ml, 10.1 ± 3.6 × 10^3^; NGF 100 ng/ml, 9.2 ± 2.2 × 10^3^; *p* > 0.05; Nonviable cells: Ctrl: 1.3 ± 0.6 × 10^3^; NGF 10 ng/ml: 3.3 ± 1.9 × 10^3^; NGF 100 ng/ml: 2.1 ± 2.6 × 10^3^; *p* > 0.05). Three independent experiments were performed in triplicate. **c** ALT, HAD, LDH, CS and GAPDH enzyme activities performed in L6C5 myoblasts grown in serum-free medium and treated with NGF for 48 h. Data are shown as fold increase with respect to their control and expressed in percent values. One unit of enzymatic activity was defined as the amount of enzyme that forms 1 µmol of product per minute per mg of tested protein. The values have been collected from three independent experiments, each performed in triplicate. *p < 0.05. **d** Analysis of JNK activation on L6C5 myoblasts treated with or without NGF under serum starvation conditions. Immunoblot analysis of phosphorylated and total JNK in L6C5 cells at different times (15, 30 and 60 min) since NGF (20 ng/ml) supplementation. The histogram represents phospho JNK/total JNK ratio as mean ± SD of experiments repeated at least three times. *p < 0.05

NGF did not affect significantly neither myoblast growth nor viability under standard culture condition (data not shown). Under serum starvation, the MTS-derived OD values of myoblasts treated with NGF 100 ng/ml were statistically higher when compared to untreated cells (*p* < 0.05) (Fig. 1a), although the Trypan blue assay (Fig. 1b) and the analysis of the apoptotic index (data not shown) excluded a significant NGF modulation of the total number of viable and nonviable cells. Since MTS quantification of viable cells depends upon the cellular metabolic rate [23], we verified the activity of some key enzymes. The results showed that, under serum deprivation, the activity of CS and GAPDH, both involved in carbohydrates metabolism, was increased by NGF supplementation compared to control (CS: 21.7 and 55.2% increase for NGF 10 and 100 ng/ml respectively; GAPDH: 37.7 and 95.7% increase for NGF 10 and 100 ng/ml respectively (*p* < 0.05) (Fig. 1c). No NGF-dependent differences were detected under standard conditions (data not shown). Previous data concerning the correlation between NGF and an increased activity in cycle Krebs enzymes also involved the activation of the JNK pathway [24]. Indeed, we demonstrated a significant enhancement of JNK phosphorylation after 15′–30′ min from NGF (20 ng/ml) supplementation in L6C5 myoblasts grown under serum starvation (*p* < 0.05) (Fig. 1d). At present, the biological significance of this result is unknown, but it agrees with published data in L6 myogenic cells showing that modification of mitochondrial homeostasis correlates with JNK phosphorylation and insulin resistance [25], opening to speculations on the role of NGF in the metabolic homeostasis of myogenic cells and possible ergogenic effect in muscle tissue [26, 27].

**Fig. 2:**
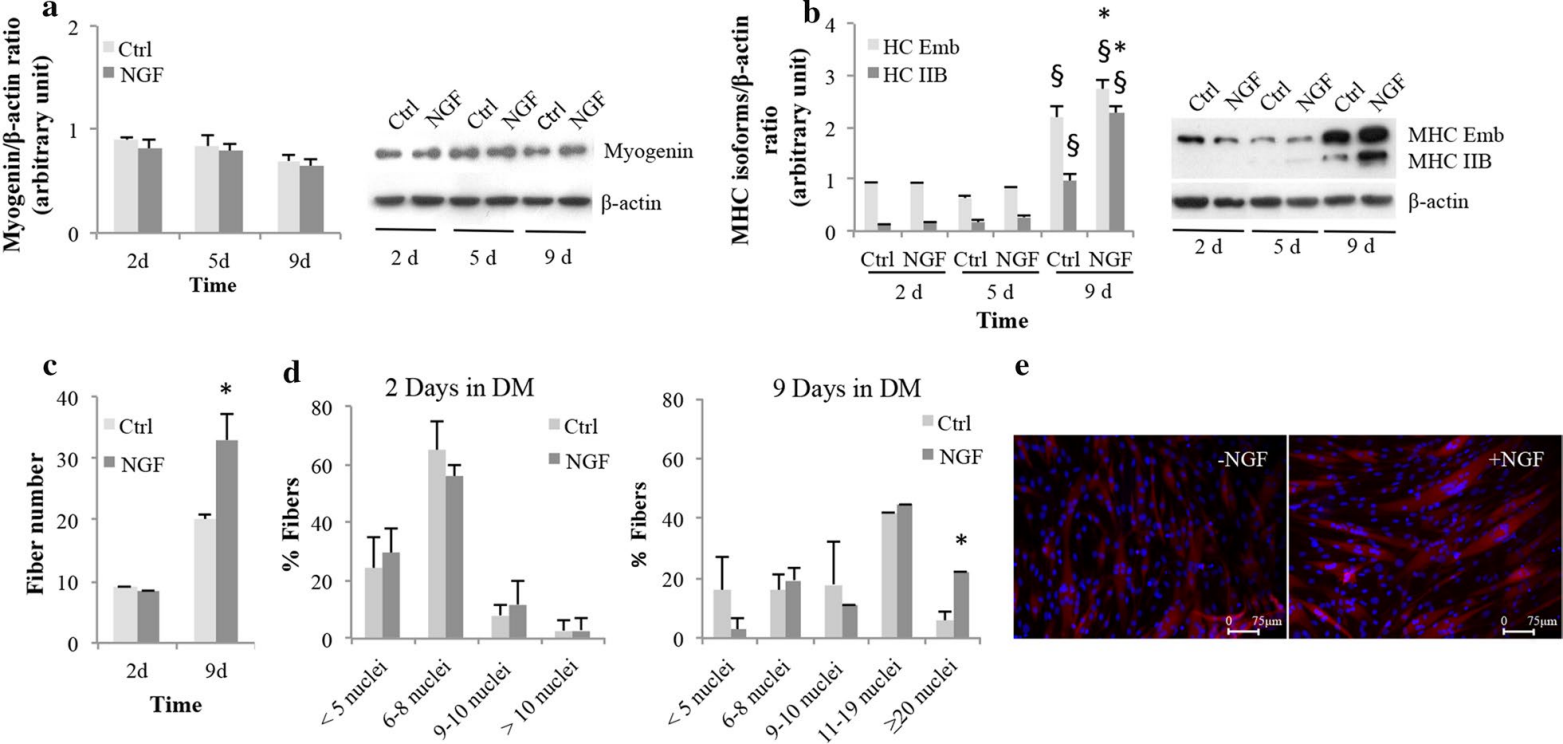
Effects of NGF supplementation on L6C5 in vitro differentiation. **a** Myogenin and **b** MyHC isoforms expression at different times of differentiation (2, 5, and 9 days post DM) (9 days: NGF MHC Emb: 2.74 ± 0.17 vs Ctrl MHC Emb: 2.19 ± 0.23; NGF MHC IIB: 2.29 ± 0.11 vs Ctrl MHC IIB: 0.98 ± 0.11; ^§^p < 0.05 vs 2 and 5 days; *p < 0.05 vs Ctrl 9d. The β-actin was used as housekeeping for both markers. **c** Analysis of fiber number during L6C5 differentiation in presence or not of NGF (NGF vs CTRL: 33 ± 4.2 vs 20 ± 1; *p* < 0.05). **d** Each histogram represents the fusion rate of L6C5 myoblasts after 2 and 9 days in DM (9 days: NGF vs CTRL: 22.2% vs 6%, *p* < 0.05). To facilitate cell analyses, the bottom of each well was divided into ten square fields and in each field the number of myotubes and nuclei/myotube were counted manually at a final magnification of ×40 (Olympus BX41 microscope, Olympus) using an ocular grid. **e** Representative images (×40) of L6C5 cells grown in DM medium for 9 days with and without NGF (20 ng/ml). Scale bar, 75 μm. The histograms represent the mean ± SD of experiments repeated at least three times. *p < 0.05

In 2011, Colombo et al. [7] proved that p75^NTR^ modulates myogenesis and dystrophin expression, proposing this receptor as a novel marker of human differentiation-prone muscle precursor cells. In agreement with Deponti et al. [3], we confirmed that NGF at the concentration of 20 ng/ml, the most effective to promote L6 myoblast proliferation and differentiation [4], did not modulate the expression of myogenin, an early myogenic marker [28], both at protein (Fig. 2a) and transcriptional level (data not shown). However, later during differentiation (9 days in DM), NGF increased both the Embrional (HC Emb) and type IIB (HC IIB) MyHC isoform contents (*p* < 0.05) (Fig. 2b). Thus, we showed for the first time that NGF, similar to growth hormone [29, 30], exerted a positive effect on the terminal markers of myogenesis [31, 32]. We also found that, at the same stage, NGF induced a significant increase in the number of fibers (*p* < 0.05) (Fig. 2c), with an excess of fibers containing more than 20 nuclei (*p* < 0.05) (Fig. 2d, e). Thus, our in vitro model confirmed a hypertrophic effect of the NGF/p75^NTR^ axis due to both increased fusion rate and increased number of fibers [6], but we also demonstrated an effect on the expression of MyHC isoforms, claiming for a putative role of NGF in the contractile properties of skeletal muscle fibers in vivo [7].

**Fig. 3:**
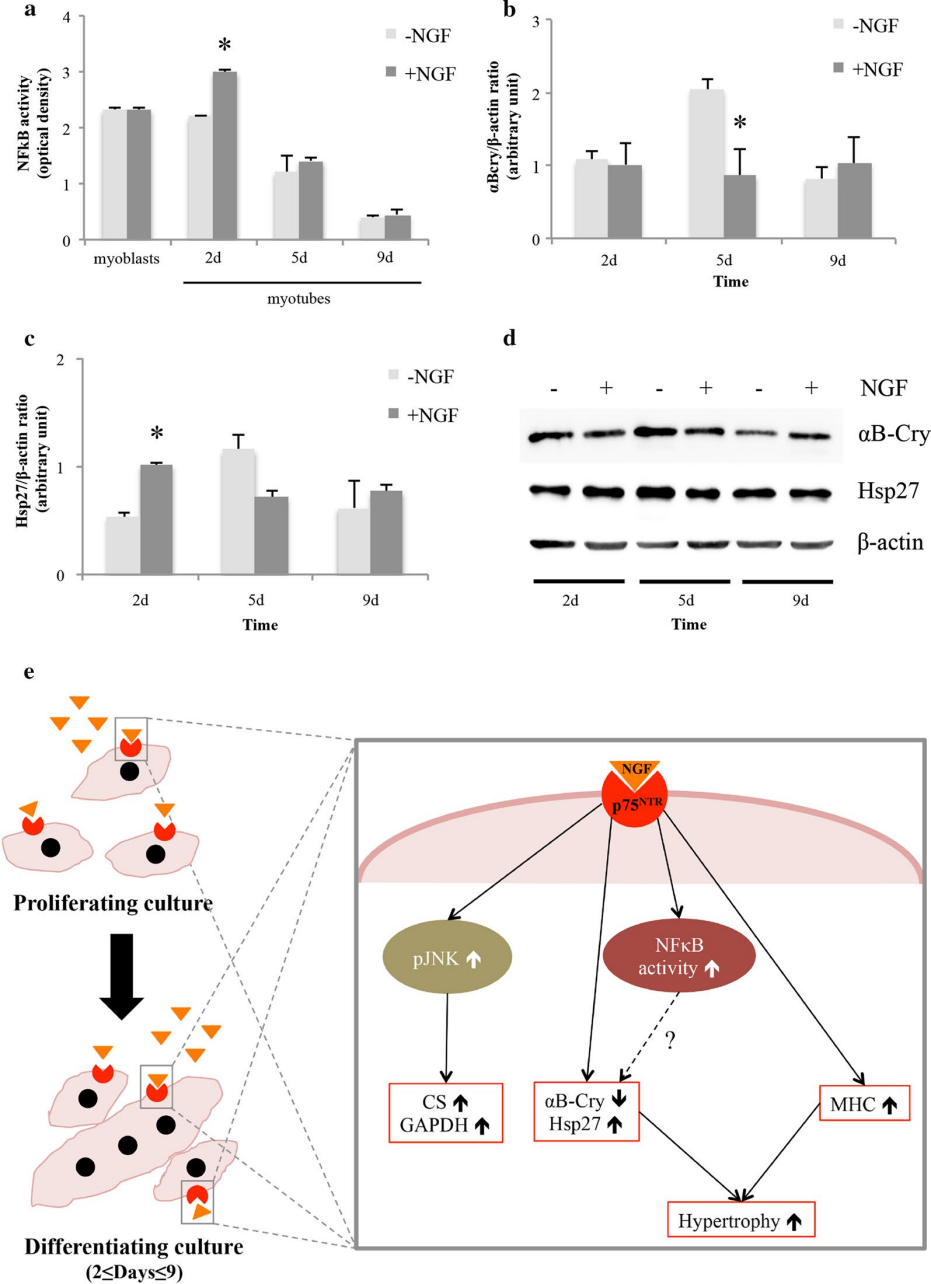
Effects of NGF supplementation on NFκB, αB-crystallin and Hsp27 during L6C5 in vitro differentiation. **a** NFκB activity measurement in myoblasts and differentiating L6C5 cells (2, 5 and 9 days) grown with or without NGF supplementation. Nuclear protein extract from Jurkat cells stimulated by TPA and calcium ionophore was used as positive control (2 days: − NGF vs + NGF, 2.2 vs 3.0, *p* < 0.05). **b, c** Quantitative analysis of αB-Cry and Hsp27 expression during differentiation process (2, 5 and 9 days) of myogenic cells supplemented with or without NGF (αB-Cry: NGF 1.3 ± 0.08 vs Ctrl 2.0 ± 0.11, *p* < 0.05; Hsp27: NGF 1.0 ± 0.03 vs Ctrl 0.53 ± 0.04, *p* < 0.05). The histograms represent the mean ± SD of experiments repeated at least three times. *p < 0.05. **d** Representative western blot of αB-crystallin and Hsp27 expression. The β-actin was used as housekeeping for both markers. **e** Proposed mechanism for NGF-p75^NTR^ signaling pathways in L6C5 myogenic cells: during myoblast proliferation and serum-deprivation condition, the supplementation with NGF can sustain the activity of key enzymes in carbohydrate metabolism, such as citrate synthase and glyceraldehyde-phosphate-dehydrogenase, through the activation of the JNK pathway. During the early stage of myoblast fusion, NGF transiently up-regulates NFκB activity and, directly or indirectly trough a NFκB-mediated mechanism, anticipates the myogenic progression by modulating αBcry and Hsp27 expression and promoting, at a late differentiation stage, myonuclear fusion and the accumulation and stabilization of the MyHC myofibrillar component. *CS* citrate synthase, *GAPDH* Glyceraldehyde-phosphate-dehydrogenase, *NGF* nerve growth factor, *αBcry* αB-crystallin, *JNK* c-Jun N-terminal kinases, *NFκB* nuclear factor kappa-light-chain-enhancer of activated B cells, *p75* ^*NTR*^ neurotrophin receptor p75

It has been recently reported that the chronic up-regulation of NFκB relates to impairment of the myogenic process in skeletal muscle or to muscle atrophy [10, 33]. Nonetheless, we and others demonstrated that the transient NFκB activation plays a role during L6C5 and C2C12 “in vitro” differentiation [17, 34], or during growth factors-promoted survival and/or differentiation of myogenic cells [9, 35]. Actually, L6C5 myogenic cultures supplemented with NGF showed a significant increase in NFκB activity compared to the control at 2 days in DM (*p* < 0.05), while no effects were detected in proliferating myoblasts (data not shown), sub-confluent myoblasts or at a later differentiation stage (Fig. 3a). This result demonstrated that, as in other cell types, also in myogenic cell line neurotrophins transiently regulate the activity of NFκB via p75^NTR^ receptor [36, 37]. We then verified in L6C5 cellular model the possible correlation between NFκB activity, modulation of sHSPs and resistance to apoptosis. Hsp27 and αB-Cry are key components of the myofibril structure, with a prominent role in skeletal muscle physio-pathology [38, 39] and in exercise-related adaptation to damaging contraction [13, 40]. We found that NGF induced a significant reduction of αB-Cry expression at 5 days in DM and an increase of Hsp27 levels after 2 days in DM (*p* < 0.05) (Fig. 3b–d). This result mirror our previous data on rat muscle regeneration in vivo [9] suggesting an anticipatory effect of NGF in the progression throughout the differentiation process, consistent with the inhibitory and promoting effects exerted during myogenic differentiation by αB-Cry and Hsp27, respectively [14, 15, 38]. However, in contrast with previous results by our group [8, 11, 17] and by others [41, 42] on the relevance of the neurotrophin/p75^NTR^ axis in the promotion of myofibres survival, in the present study we did not find any protective effects of NGF towards spontaneous or H_2_O_2_-induced apoptosis, neither in proliferating nor in differentiating L6C5 cells. Indeed, as already demonstrated in a fibroblast derived cell line constitutively expressing rat p75 [43], in our “in vitro” model NGF did not modulate neither the total number of TUNEL-positive apoptotic cells, nor the expression and/or cleavage of Bcl-2 and Caspase-3 in differentiating cells (Additional file 2: Fig. S2) and in myoblasts (data not shown).

As summarized in Fig. 3e, our results demonstrate that p75^NTR^ is sufficient to mediate the NGF modulation of L6C5 cells differentiation inducing structural, metabolic and functional changes as well as NGF direct, or indirect effect on αB-Cry and Hsp27, essential for the myogenic program and myofibrillar stabilization. Moreover, we show that the NGF-mediated hypertrophic at the late stage of differentiation correlates to an increased expression of MyHC isoforms.

## Limitations

Although the results on the NGF-mediated effects on the enzymatic activities, NFκB activation and sHSPs expression are solid, the study did not reveal the causal relationship among these factors.

## Additional files

**Additional file 1: Figure S1.** TrkA and p75^NTR^ expression in L6C5 myoblasts and myotubes. **a**, **b** Relative mRNA levels of TrkA and p75^NTR^ in L6C5 cells at different times since the seeding (proliferating = 24, 48 h; 2, 5, 9, and 12 days of differentiation). Proliferating (Pr) and differentiated (Dif) PC12 cells were used as positive control for the expression of TrkA and p75^NTR^ receptors. **c** Western blot analysis of TrkA and p75^NTR^ in proliferating and differentiated L6C5 cells. RNA extraction and quantitative RT-PCR was performed as already described [44]. Primers for PCR amplification were as follows: housekeeping gene glyceraldehyde-3-phosphatedehydrogenase (GAPDH): 5′-ACCACAGTCCATGCCATCAC-3′ and 5′-TCCACCACCCTGTTGCTGTA-3′; Neurotrophic tyrosine kinase receptor type 1 (TrkA): 5′-CCTGATGCCTTCCATTTCAC -3′ and 5′-TGACATTGACCAGAGTTAGCC-3′; Nerve growth factor receptor (p75^NTR^): 5′-CAAGGAGACATGTTCCACAG-3′ and 5′GGATCTCTTCGCATTCAGCA-3′. *L6C5-P* proliferating myoblasts in GM, *L6C5-D* differentiating cultures in DM.

**Additional file 2: Figure S2.** Effect of NGF supplementation on spontaneous or H_2_O_2_-induced apoptosis during L6C5 in vitro differentiation. **a** TUNEL assay (Roche applied sciences) and **b** Bcl-2 and Caspase-3 protein expression in L6C5 cells growing in DM NGF-supplemented under standard and oxidative stress condition (100 μM H_2_O_2_). For the analysis of H_2_O_2_-induced apoptosis, cells under differentiation (48 h before, or 2, 5 or 9 days from DM addiction) in presence or in absence of NGF (20 ng/ml) were treated with H_2_O_2_ 100 µM for the last 1-h of culture. The histogram represents the mean ± SD of experiments repeated at least three times. *p < 0.05 compared with control (Ctrl). ^§^ *p* < 0.05 compared with control NGF-supplemented (Ctrl + NGF).

## Abbreviations

ALT: alanine transglutaminase
αB-Cry: αB-crystallin
CS: citrate synthase
DMEM: Dulbecco modified eagle medium
DM: differentiation medium
GAPDH: glyceraldehyde-phosphate-dehydrogenase
GH: growth hormone
GM: growing medium
HAD: 3-OH acylCoA dehydrogenase
JNK: c-Jun N-terminal kinase
LDH: lactate dehydrogenase
MTS: 3-(4,5-dimethylthiazol-1)-5-(3-carboxymeth-oxyphenyl)-2-(4-sulfophenyl)-2H-tetrazolium), inner salt
MyHC: myosin heavy chain
NFκB: nuclear factor κB
NGF: nerve growth factor
p75^NTR^: p75 neurotrophin receptor
sHSPs: small heat shock proteins
TrkA: tyrosin kinase receptor A
TPA: 12-otetradecanoylphorbol-13-acetate
