# Supplementary figures and images for: The p75NTR-mediated effect of nerve growth factor in L6C5 myogenic cells
Source: BMC Res Notes. 2017 Dec 4;10:686. doi: 10.1186/s13104-017-2994-x (PMC5716223; doi:10.1186/s13104-017-2994-x)

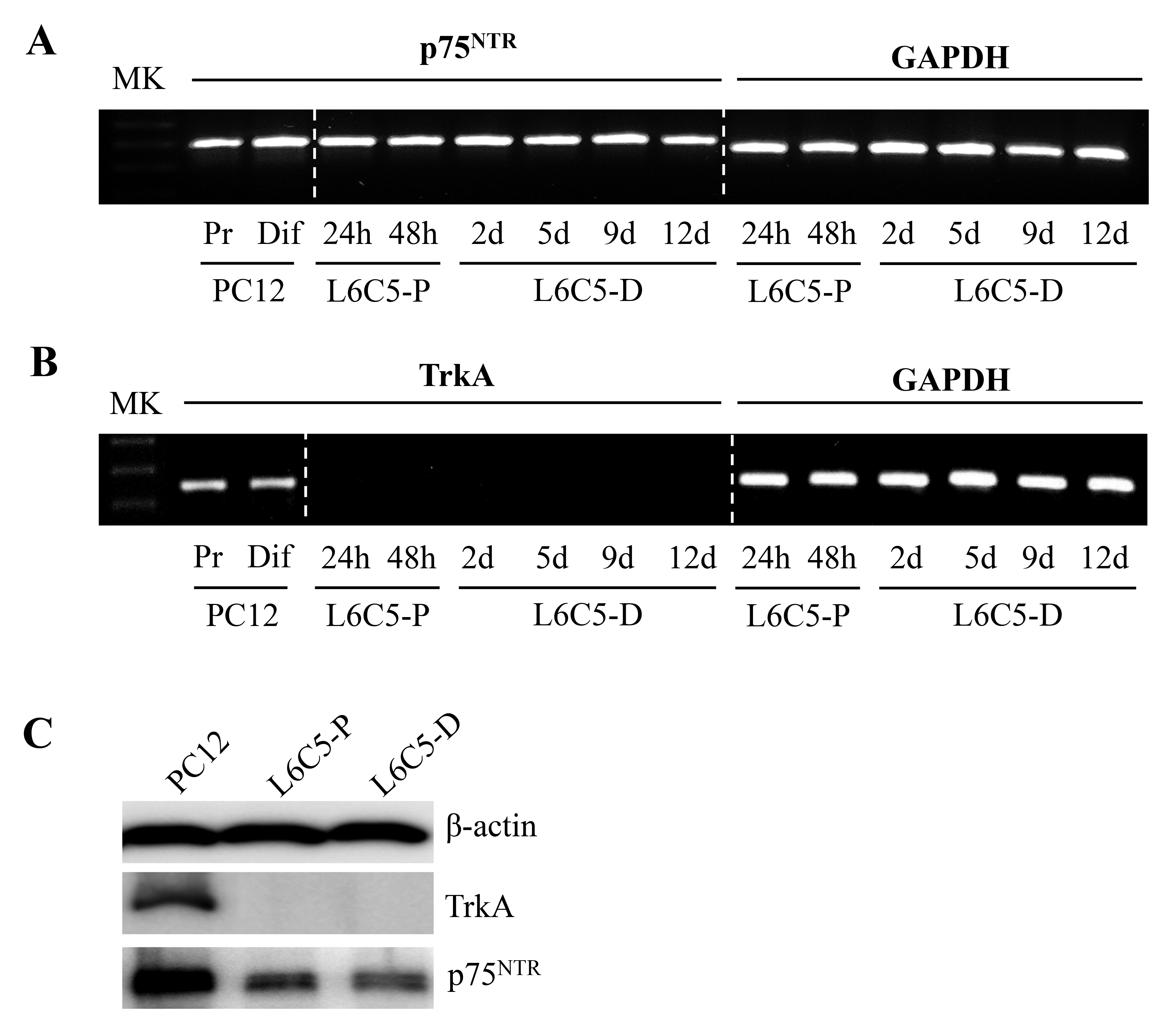

Supplement: Supplementary file 1 — Additional file 1: Figure S1. TrkA and p75NTR expression in L6C5 myoblasts and myotubes. a, b Relative mRNA levels of TrkA and p75NTR in L6C5 cells at different times since the seeding (proliferating = 24, 48 h; 2, 5, 9, and 12 days of differentiation). Proliferating (Pr) and differentiated (Dif) PC12 cells were used as positive control for the expression of TrkA and p75NTR receptors. c Western blot analysis of TrkA and p75NTR in proliferating and differentiated L6C5 cells. RNA extraction and quantitative RT-PCR was performed as already described [44]. Primers for PCR amplification were as follows: housekeeping gene glyceraldehyde-3-phosphatedehydrogenase (GAPDH): 5′-ACCACAGTCCATGCCATCAC-3′ and 5′-TCCACCACCCTGTTGCTGTA-3′; Neurotrophic tyrosine kinase receptor type 1 (TrkA): 5′-CCTGATGCCTTCCATTTCAC -3′ and 5′-TGACATTGACCAGAGTTAGCC-3′; Nerve growth factor receptor (p75NTR): 5′-CAAGGAGACATGTTCCACAG-3′ and 5′GGATCTCTTCGCATTCAGCA-3′. L6C5-P proliferating myoblasts in GM, L6C5-D differentiating cultures in DM. [file 13104_2017_2994_MOESM1_ESM.jpg]

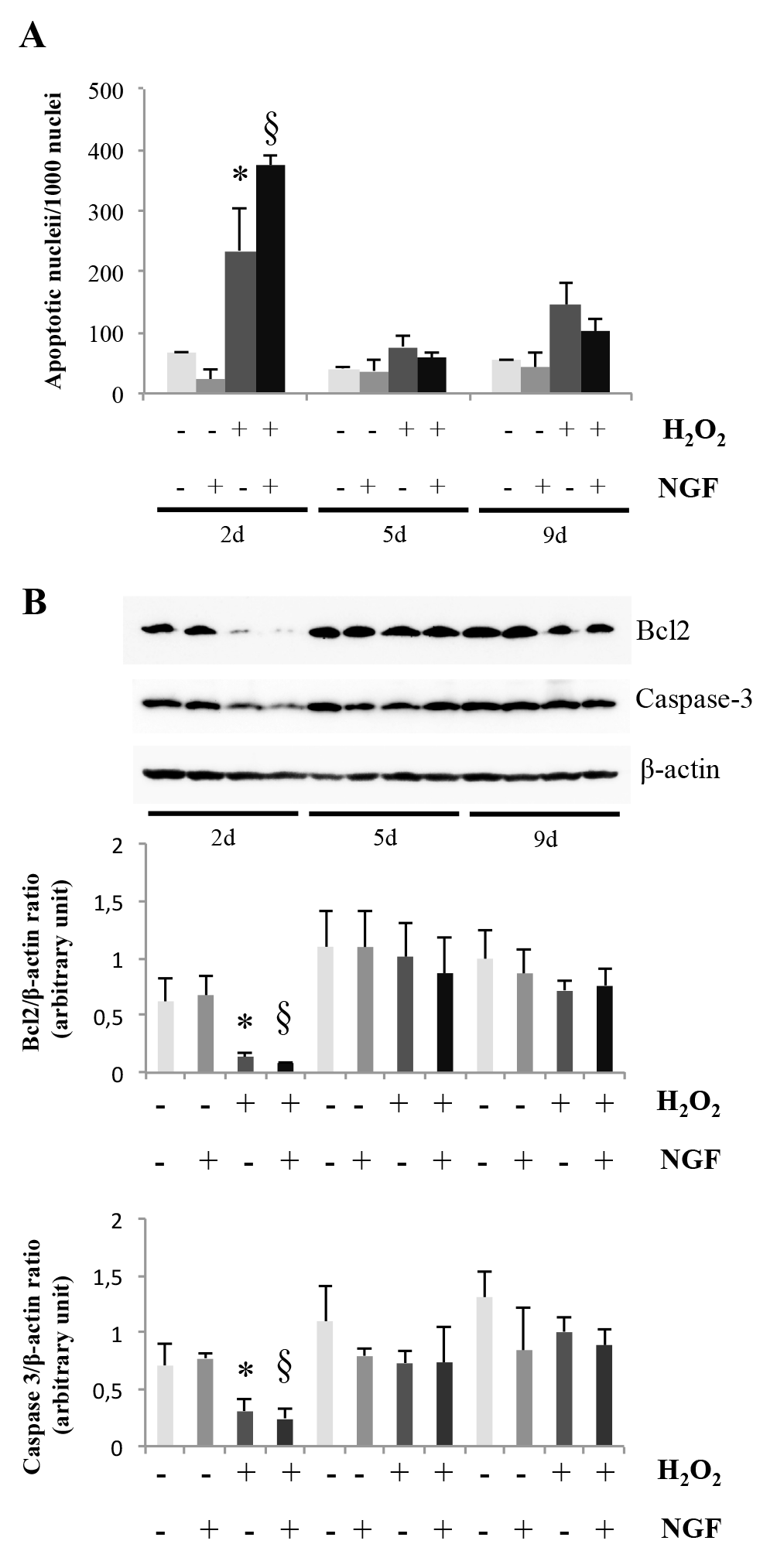

Supplement: Supplementary file 2 — Additional file 2: Figure S2. Effect of NGF supplementation on spontaneous or H2O2-induced apoptosis during L6C5 in vitro differentiation. a TUNEL assay (Roche applied sciences) and b Bcl-2 and Caspase-3 protein expression in L6C5 cells growing in DM NGF-supplemented under standard and oxidative stress condition (100 μM H2O2). For the analysis of H2O2-induced apoptosis, cells under differentiation (48 h before, or 2, 5 or 9 days from DM addiction) in presence or in absence of NGF (20 ng/ml) were treated with H2O2 100 µM for the last 1-h of culture. The histogram represents the mean ± SD of experiments repeated at least three times. *p < 0.05 compared with control (Ctrl). § p < 0.05 compared with control NGF-supplemented (Ctrl + NGF). [file 13104_2017_2994_MOESM2_ESM.tif]
